# Differential Viral Genome Diversity of Healthy and RSS-Affected Broiler Flocks

**DOI:** 10.3390/microorganisms10061092

**Published:** 2022-05-25

**Authors:** Jakub Kubacki, Weihong Qi, Cornel Fraefel

**Affiliations:** 1 Institute of Virology, Vetsuisse Faculty, University of Zurich, 8057 Zurich, Switzerland; cornel.fraefel@uzh.ch; 2 Functional Genomics Center Zurich, 8057 Zurich, Switzerland; weihong.qi@fgcz.ethz.ch

**Keywords:** poultry virome, next-generation sequencing, runting-stunting syndrome, broiler flocks

## Abstract

The intestinal virus community contributes to health and disease. Runting and stunting syndrome (RSS) is associated with enteric viruses and leads to economic losses in the poultry industry. However, many viruses that potentially cause this syndrome have also been identified in healthy animals. To determine the difference in the virome of healthy and diseased broilers, samples from 11 healthy and 17 affected broiler flocks were collected at two time points and analyzed by Next-Generation Sequencing. Virus genomes of *Parvoviridae*, *Astroviridae*, *Picornaviridae*, *Caliciviridae*, *Reoviridae*, *Adenoviridae*, *Coronaviridae*, and *Smacoviridae* were identified at various days of poultry production. De novo sequence analysis revealed 288 full or partial avian virus genomes, of which 97 belonged to the novel genus *Chaphamaparvovirus*. This study expands the knowledge of the diversity of enteric viruses in healthy and RSS-affected broiler flocks and questions the association of some viruses with the diseases.

## 1. Introduction

Poultry is a major source of animal protein for human consumption worldwide. The need for high productivity, low feed quality, poor flock management, i.e., high animal density, and insufficient environmental and pathogen control compromises animal health [1]. The gastrointestinal microbiome and virome, in particular, have a major impact on health and disease [2]. While many studies have explored the virome of humans and various animal species, that of avian species is largely unknown [3,4].

Of specific interest in poultry health management is runting and stunting syndrome (RSS), also known as malabsorption syndrome. Although the disease has been known for more than 40 years, it is unclear which viruses are implicated in the disease, since many different RNA and DNA viruses have been detected in affected birds [5]. Clinical signs of RSS include poor feed conversion rate (FCR), uneven growth of individual animals within the flock, decreased body weight, poor feather development, diarrhea, lameness, and high mortality [6].

Previous studies to associate specific viruses with RSS concluded that the disease is polyetiological with the involvement of several different viruses, including avian nephritis virus (ANV), astrovirus (CAstV), parvovirus, rotaviruses (RVs), and avian orthoreovirus (ARVs) [7]. Recently, picornaviruses and calicivirus have been detected in RSS-affected chickens as well [8]. However, these prior studies have mainly investigated diseased animals, while the normal viral community of healthy birds and how it varies among populations and over time remain unexplored. Currently, next-generation sequencing (NGS) is extensively used in virus discovery in humans and livestock, enabling unbiased detection of enormous viral sequence datasets [9,10]. The aim of our study was to use NGS to determine the differential virus community of healthy broiler flocks and broiler flocks affected by RSS.

## 2. Materials and Methods

### 2.1. Samples

Samples from broiler flocks (Ross 308) were collected twice during production time by pulling cloth material over the floor of the breading facility. In healthy animal flocks, the first sample was taken between days 2 and 11 (mean 4.9) and the second between days 30 and 35 (mean 33.3) of production. In RSS animal flocks, the first sample was taken at the onset of symptoms, i.e., between days 7 and 31 (mean 18.6), and the second between days 27 and 36 (mean 33.8) of production. The cloth material from healthy (*n* = 11) and diseased broiler flocks (*n* = 17) was placed individually into zip-lock bags and, after adding 30 mL of PBS and mixing/squeezing, the liquid from each bag was transferred into a 50 mL tube. Then, 10 mL of liquid was transferred to an Amicon^®^ Ultra-15 Centrifugal Filter (Merck Millipore Ltd., Burlington, MA, USA) and centrifuged for 20 min at 4000 rpm. Five-fold concentrated supernatant was recovered, transferred to a new tube, and centrifuged for 5 min at 16,000× *g*.

### 2.2. Sample Preparation and Sequencing

As a high abundance of cellular nucleic acids may compromise virus detection, a protocol previously established to enrich virus particles was applied to increase recovery of viral genetic material and to achieve sufficient depth and sequence diversity that supports constructing virus genomes [11]. Enrichment of viral nucleic acids was followed by reverse transcription for RNA viruses and sequence-independent single primer amplification for total DNA. Sequencing libraries were diluted to 50 ng of DNA sheared to 500 bp length using the E220 Focused-ultrasonicator (Covaris, Woburn, MA, USA) and prepared with the NEBNext^®^ Ultra™ II DNA Library Prep Kit for Illumina^®^ (New England Biolabs, Ipswich, MA, USA) according to the manual. The library pool was sequenced at the Functional Genomics Center Zurich (FGCZ) in a paired-end 2 × 150 bp, SP flow cell sequencing run using the NovaSeq 6000 (Illumina, San Diego, CA, USA). The PhiX Control v3 Library (Illumina, San Diego, CA, USA) was used as the control.

### 2.3. Data Analysis

The sequences were analyzed by de novo assembly and reference-guided in-house assembly pipelines [12,13]. First, Illumina sequencing adapters (default) and low-quality sequencing ends were trimmed using Trimmomatic (v0.39) [14]. The 5′-end of each read was quality clipped using a sliding window trimming approach, where the end was clipped if the average quality within the 4 bp window fell below a threshold of 15. On the 3′-end, bases below quality 20 were clipped. Second, trimmed reads with average quality above 20 and length longer than 40 nt were further cleaned up by trimming away the SISPA primer using cutadapt (v3.2, “-b GTTGGAGCTCTGCAGTCATC -B GTTGGAGCTCTGCAGTCATC”). Subsequently, quality-checked reads were assembled using metaspades (v3.12.0) [15]. Assembled de novo contigs were compared to the NCBI non-redundant database (nt) using blastn (v2.10.1+) and annotated using the best blastn hits. Quality-checked reads were mapped back to the assembled sequences using bwa (v0.7.17) mem [16]. Alignment summary statistics per contig were collected by running samtools (v1.11) idxstats. Additionally, to detect viruses with low read numbers (cut off 3 reads over the genome), trimmed reads were aligned in a metagenomic pipeline of the SeqMan NGen v.17 (DNAStar, Lasergene, Madison, WI, USA) to an in-house database containing 60,000 full-length viral genomes downloaded from the NCBI database and visualized in the SeqMan Ultra (DNAStar, Lasergene, Madison, WI, USA).

### 2.4. Phylogenetic Analysis

The viral contigs and selected coding regions were further investigated and aligned using MUSCLE in the MEGA X Clone manager ver. 9 (Sci-Ed) and Sequence Demarcation Tool Version 1.2 (SDTv1.2) [17]. Phylogenetic trees were constructed in Mega X using the Maximum Likelihood algorithm with 1000 bootstrap values and a cut-off of 70% [18]. The nucleotide sequences used for phylogenetic analysis were named with a pattern “PBX-H/SI/IIY” (X, sample number; H, healthy flock; S, RSS-affected flock, I/II, first/second sampling time point; Y, the day of production).

### 2.5. Accession Numbers

The nucleotide sequences of viruses from this study shown in the phylogenetic analysis have been registered at GenBank under the following accession numbers: OM469021-OM469308. All raw sequencing data generated were uploaded to the Sequence Read Archive (SRA) under accession number PRJNA802076.

## 3. Results

The total number of raw sequencing reads generated from all 56 flock samples was 5.69 × 10^8^ (mean of 1.02 × 10^7^ sequencing reads per sample; range 4.2 × 10^6^–2.88 × 10^7^ reads per sample). Through reference-based alignment, viral reads from 8 virus families were identified: *Parvoviridae*, *Astroviridae*, *Picornaviridae*, *Caliciviridae*, *Reoviridae*, *Adenoviridae*, *Coronaviridae*, *Smacoviridae,* and unknown viral family. BLASTn revealed 1070 contigs with lengths > 1000 nt constructed by de novo assembly.

### 3.1. Viral Community

While seven of the 8 virus families were identified in both healthy and RSS flocks, sequences from *Coronaviridae* were detected only in one RSS flock at the end of production (day 34). In healthy flocks, the rate of the normalized mean sequencing reads with a viral origin (all viral sequencing reads divided by the total raw reads generated within the sample) increased from 0.5% (range: 0.0003–1.92%) to 0.62% (range: 0.09–5.43%) from the first to the second sampling time point. In RSS-affected broiler flocks, the same rate decreased from 1.38% (range: 0.26–8.31%) to 0.75% (range: 0.09–7.77%) between the first (onset of symptoms) and the second sampling time point. The most abundant virus family in healthy bird flocks was *Astroviridae* at the first time point and *Parvovirdae* and *Picornaviridae* at the second time point (Table 1 and Table 2). For the RSS flocks, *Parvoviridae*, followed by *Picornaviriade*, were the most abundant virus families at both time points. Interestingly, at days 2–4 of production, very few viral reads were detected, while on days 5, 6, and 11 in healthy broiler flocks and days 7 and 14 in RSS flocks, the highest abundance of *Astrovirdae* within both timepoints and group types was identified (Table 2).

### 3.2. Parvoviridae

**Figure 1:**
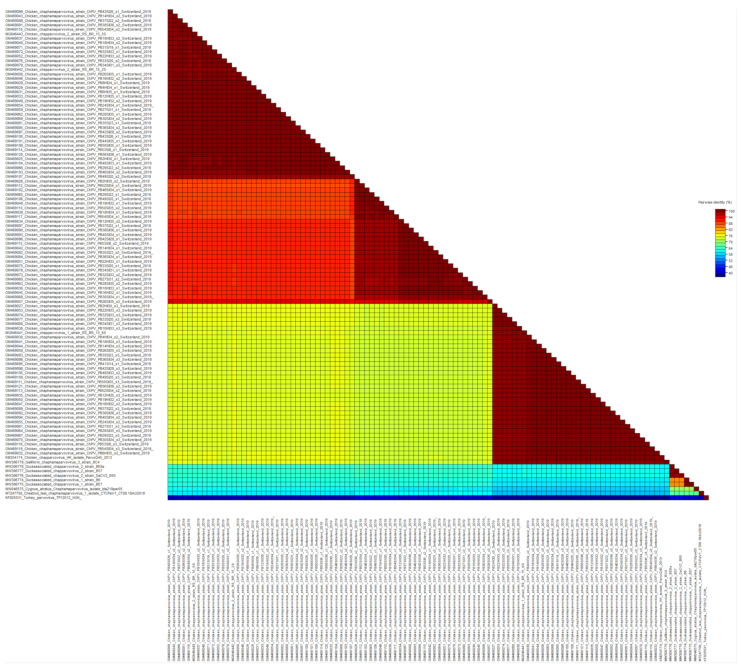
Color-coded matrix of pairwise sequence identity scores of the chaphamaparvoviruses NS1 protein generated with the Sequence Demarcation Tool ver. 1.2 (SDTv1.2).

**Figure 2:**
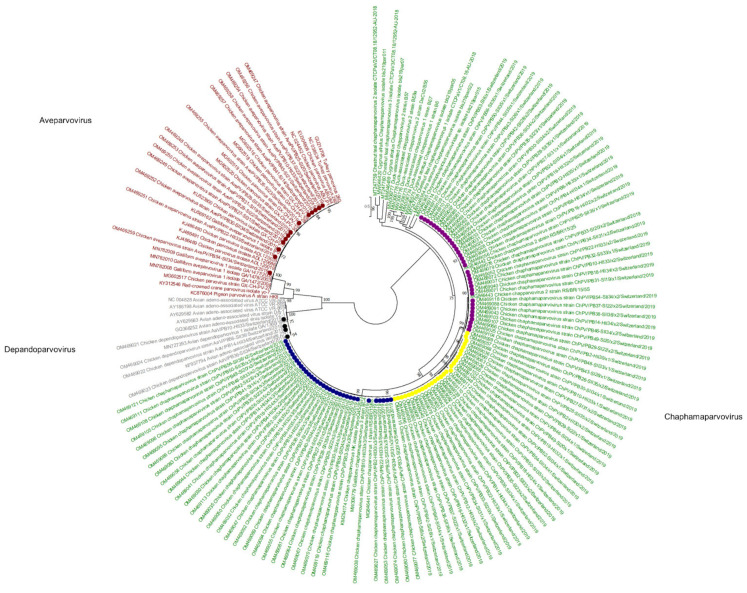
Phylogenetic tree based on amino acid sequence identity of the NS1 protein of selected parvoviruses. In red, gray, and green, the genera of *Aveparvovirus*, *Depandoparvovirus*, and *Chaphamaparvovirus*, respectively, are presented. The *Aveparvovirus* contigs constructed in this study are marked by red dots. The *Depandopavovirus* contigs constructed in this study are marked by black dots. Blue and yellow dots show separate branches of chaphamaparvovirus 1 (aa identity of the polyprotein between both branches is above 85%). Pink dot, chaphamaparvoviruses 2. Phylogenetic analysis was performed using the maximum likelihood algorithm based on the Tamura–Nei model with the 1000 replication bootstrap method. Only values ≥ 70% were displayed. Evolutionary analyses were conducted using MEGA X.

Viral reads matching parvoviruses were detected in 55/56 samples. In de novo assembly, 114 viral contigs belonging to *Parvoviridae* were constructed, annotated, and submitted to GenBank (Figure 1). At the amino acid (aa) level of NS1, 97/114 reads belong to the subfamily *Hamaparvovirinae* and the genus *Chaphamaparvovirus*. Of these 33/97 were assigned to the species *Galliform chaphamaparvovirus 3*, and 64/97 were assigned to the species *Galliform Chaphamaparvovirus 2*. Of the latter, 35/64 have 98–100% NS1 aa identity, and 29/64 have 88–90% NS1 aa identity to strains from Brazil (MG846442 and MG846443). According to the ICTV, parvoviruses that share at least 85% NS1 protein sequence identity are classified into one species. However, the color-coded matrix of sequence identity generated with the SDT revealed two subgroups within the species *Chaphamapravovrises 2*, and the two groups formed separate clusters on the phylogenetic tree as well (Figure 1 and Figure 2). Interestingly, 75 of the 97 *Chaphamaparovirus* genomes were assembled from 25 samples, meaning that some flocks harbored up to 3 different strains. *Chaphamapravovirus* contigs were identified in all 11 healthy and all 17 RSS flocks at the end of production, and in 9 RSS flocks already at the first collection time point.

Four of the 114 viral contigs, which were identified in 2 healthy flocks and 2 RSS flocks at the end of production, were assigned to the subfamily *Parvovirinae*, genus *Depandoparvovirus*. On the aa level of the Rep protein, the contigs showed 98–99% identity to other depenadoparvoviruses detected in broiler samples in China, the USA, and Russia (GQ368252, AY629583, MN727393, and KF937794).

Finally, 13/114 *Parvoviridae* contigs were assigned to the subfamily *Parvovirinae* in the genus *Aveparvovirus*. Contigs were identified in 8 RSS flocks at the first time point of collection (days 14–31), in 3 RSS flocks at the second time point (days 34–35), and in 2 healthy flocks at the end of production (both days 33). On the aa level of the NS1 protein, the contigs showed 99% identity with strains from China and South Korea (MG602518-MF602520, KJ486489-91). However, most of the constructed contigs cover only the NS1 and NP1 proteins, while VP1 and VP2 capsid proteins are missing. In one healthy flock (sample PB10) at the end of production (day 33) genomes of all 5 parvoviruses, i.e., 3 strains of *Chaphamapravovirus*, 1 *Depandoparvovirus*, and 1 *Aveparvovirus* were detected.

### 3.3. Astroviridae

**Figure 3:**
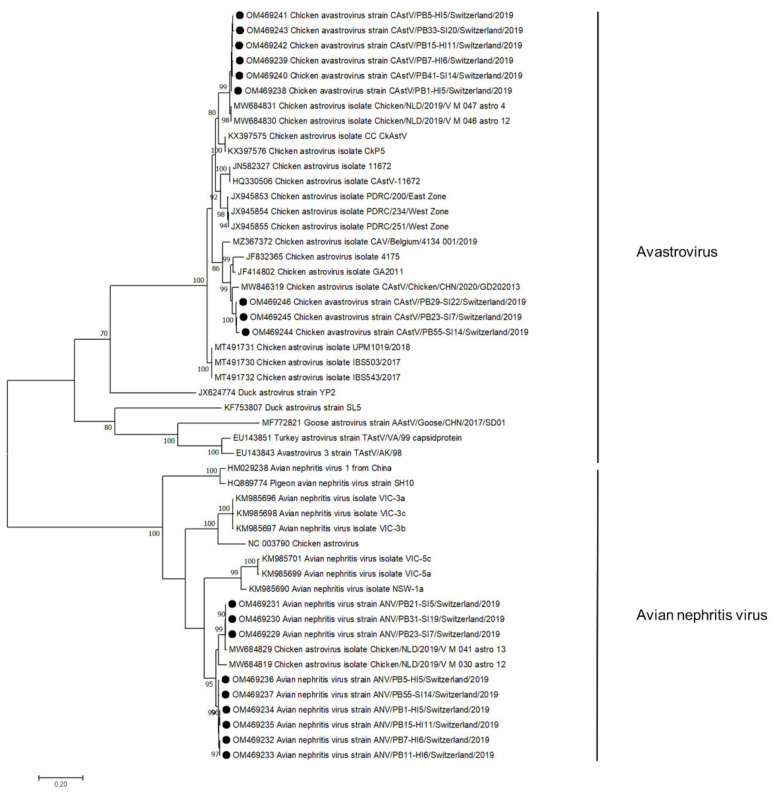
Phylogenetic tree based on the amino acid sequence identity of the capsid protein of avian avastroviruses and avian nephritis viruses. Genomes identified in this study are marked with black dots. Phylogenetic analysis was performed using the maximum likelihood algorithm based on the Tamura–Nei model with the 1000 replication bootstrap method. Only values ≥ 70% were displayed. Evolutionary analyses were conducted using MEGA X.

Sequences aligned to the family *Astroviridae* were detected in 50/56 samples. In de novo assembly, 18 astrovirus contigs were constructed, annotated, and submitted to GenBank (Figure 3). In 3 samples, viral contigs were assigned to the CAstV, in 3 to ANV, and in 6 samples, contigs of both viruses were constructed. CAstV contigs were constructed in samples from both healthy (*n* = 4, days 5–11) and RSS flocks (*n* = 5, days 7–22) at the first collection time point. In 3 RSS samples, the CAstV capsid protein showed 95% aa identity to strains from China and the USA (MW846319, JF414802). The other 6 astrovirus contigs showed 98% capsid aa identity to sequences detected in broiler feces in the Netherlands (MW68430). ANV contigs were constructed in both healthy (*n* = 6, days 5–11) and RSS flocks (*n* = 3, days 7–19). All sequences showed 95% aa identity to a strain from the Netherlands (MW684829).

### 3.4. Picornaviridae

**Figure 4:**
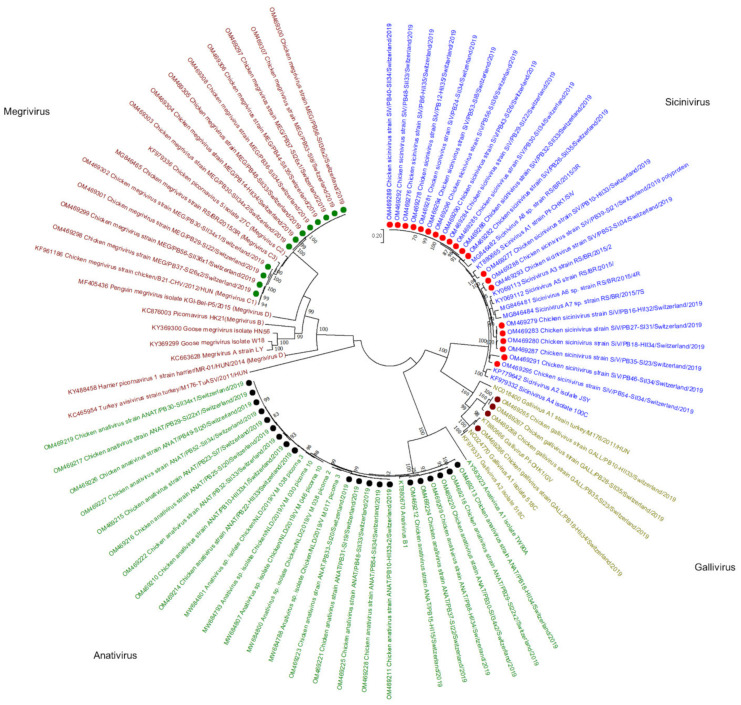
Phylogenetic tree based on the amino acid sequence identity of the polyproteins of selected picornaviruses. In red, green, olive, and blue, *Megrivirus*, *Anativirus*, *Gallivirus*, and *Sicinivirus* genera are presented, respectively. Contigs constructed in this study are marked by green (*Megrivirus*), black (*Anativirus*), purple (*Gallivirus*), and red (*Sicinivirus*) dots. Phylogenetic analysis was performed using the maximum likelihood algorithm based on the Tamura–Nei model with the 1000 replication bootstrap method. Only values ≥ 70% were displayed. Evolutionary analyses were conducted using MEGA X.

Viral reads assembled to the *Picornaviridae* family were detected in 43/56 samples. In de novo analysis, 57 viral contigs matching *Picornaviridae* were constructed, annotated, and submitted to the GenBank, of which 21 were assigned to the genus *Sicinivirus*, 20 to genus *Anativirus*, 12 to *Megrivirus,* and 4 to *Gallivirus* (Figure 4). *Sicinivirus* contigs were constructed in 5 healthy broiler flocks (days 32–35) at the end of production, in 6 RSS-affected flocks at the first sampling time point (days 8–31), and in 10 RSS flocks at the second sampling time point (33–36). Based on the polyprotein aa identity, one of the *Sicinivirus* genomes identified in this study showed 90% identity to strain A1 (KT880665), four showed 85–89% identity to A5 (KY069112), four 90% to A3 (KY069113), and twelve 91–92% to A8 (MG846482).

*Megrivirus* contigs were constructed in one healthy flock sample at the second time point of collection (day 34). In RSS-affected animals, *Megrivirus* contigs were identified in 3 flocks at the first collection time point (days 8–26) and in 5 flocks at the second (days 33–36). Moreover, in 3 flocks, two different *Megrivirus* strains were identified that clearly clustered separately. In total, all *Megrivirus* genomes from this study were clustered together with *Megrivirus C* species, of which 4 are closely related to C1 with 98% polyprotein aa identity to a Hungarian strain (KF961186); 8 genomes clustered in a separate branch with 97% identity to the Hungarian strain.

*Anativirus* contigs were constructed from reads obtained from 17 flocks, in 3 of which two genome variants were built. In total, 5 *Anativirus* contigs were constructed in 4 healthy broiler flocks at the end of production (days 33–34) and one at the first sampling time point (day 11). In RSS-affected animals, 8 contigs were constructed in 7 flocks at the first collection time point (days 7–22) and 6 contigs in 5 flocks at the second collection time point (days 33–34). All 20 *Anativirus* contigs belong to the B1 species with approximately 95% polyprotein aa identity to a reference strain (KT880670).

*Gallivirus* species A contigs were constructed in 4 samples, of which 2 were from healthy flocks from the second sampling time point (days 33–34) and two from RSS flocks at the first (day 23) and the second (day 35) time point of collection. One contig showed 96% polyprotein aa similarity to gallivirus A2 (KT880666), while 3 contigs showed only 69% polyprotein aa identity to the same strain.

### 3.5. Reoviridae

The most differential abundance of viral reads between healthy and RSS flocks concerned the *Reoviridae*. In healthy flocks, only 484 viral reads were assigned to *Reoviridae*, of which 369 reads were detected at the second time point of collection. In RSS flocks, 51,061 viral reads were assigned to either RVAs (at both collection time points) or ARVs (at the first time point). These included two RVA genomes with 10/11 (day 13) and 11/11 (day 33) segments covered, respectively, one RVF genome with 5/11 segments (day 36), and 7 ARV genomes with between 7/10 to 10/10 segments; 6 genomes were detected at the first collection time point (day 14–20) and in 1 genome at the second collection time point (day 34). All *Reoviridae* genomes detected here were submitted to GenBank. 

Analysis of two RVA sequences detected by the algorithm used to determine RVA segment specific genotypes (https://www.viprbrc.org/, accessed on 28 March 2022), showed that both are of G19P [30] genotype and have a R6-C6-M7-I11-A16-N6-T8-E10/X-R6 backbone constellation for VP1, VP2, VP3, VP6, NSP1, NSP2, NSP3, NSP4, and NSP5, respectively (Table 3). Genotyping for RVF could not be performed since only the VP1, VP2, VP3, VP6, and NSP2 genes were partially or fully sequenced. Although there are not many fully sequenced chicken RVF sequences available in GenBank, based on the VP2 gene, the RVF sequences identified in this study show 85% nt identity to the RVF strain 03V0568 from Germany (JQ919995).

**Figure 5:**
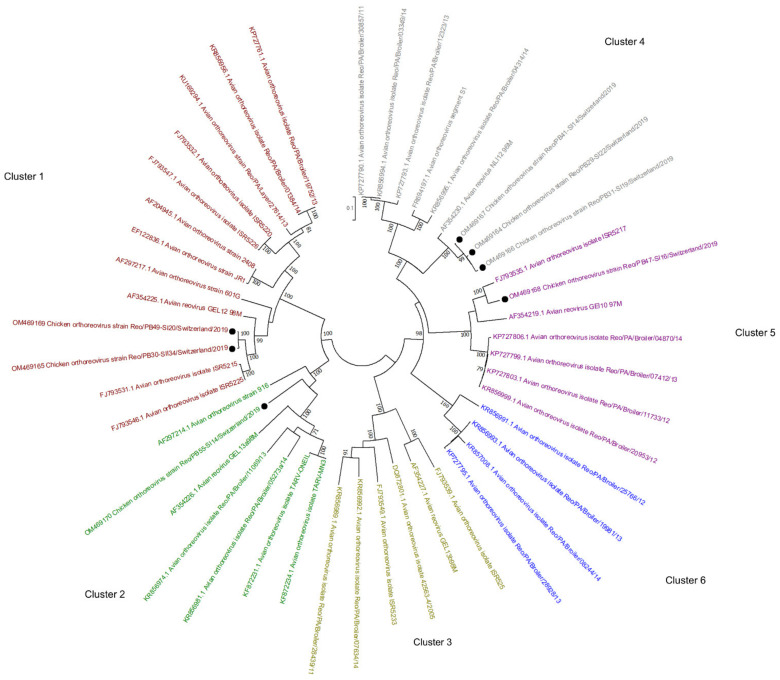
Phylogenetic tree based on the σC aa identity of selected avian orthoreoviruses. The tree shows 6 genotyping clusters in red, green, olive, blue, purple, and gray for Clusters 1, 2, 3, 6, 5, and 4, respectively. Sequences obtained in this study are marked with black dots. Phylogenetic analysis was performed using the maximum likelihood algorithm based on the Tamura–Nei model with the 1000 replication bootstrap method. Only values ≥ 70% were displayed. Evolutionary analyses were conducted using MEGA X.

The phylogenetic tree based on the sigma C gene revealed that one of the ARV genomes detected here belongs to cluster 1 (OM469165 and OM469169), one to cluster 2 (OM469170), three to cluster 4 (OM469164, OM469166, OM469167), and one to cluster 5 (OM469168) (Figure 5). In one flock, ARV contigs belonging to cluster 4 and cluster 1 were identified at the first and second sampling time points, respectively.

### 3.6. Calicivirdae

**Figure 6:**
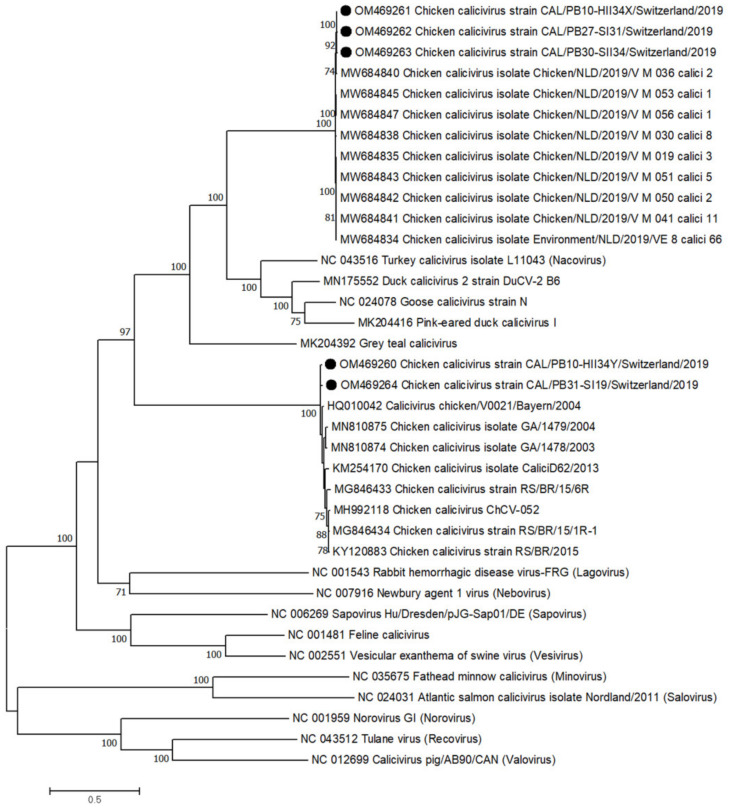
Phylogenetic tree based on the amino acid sequence identity of the polyprotein of reference strains from the genus *Calicivirus*, most closely related to the contigs from this study (black dots). Phylogenetic analysis was performed using the maximum likelihood algorithm based on the Tamura–Nei model with the 1000 replication bootstrap method. Only values ≥ 70% were displayed. Evolutionary analyses were conducted using MEGA X.

In total, 5 calicivirus contigs were constructed in 4 flocks, of which 2 contigs came from the same healthy flock (second collection, day 34) and 3 from RSS flocks from the first (days 18 and 31) and second (day 34) collection time points. Two contigs showed 98.8% polyprotein aa identity to a Bavarian strain (HQ010042) and clustered in the genus *Bavovirus*. Three contigs showed approximately 99% polyprotein aa identity to a strain from the Netherlands (MW684845) and clustered together with 9 other strains from the Netherlands in the same, yet unnamed, branch (Figure 6). The similarity between the two genomes clustering in the *Bavovirus* genus was 97%, while the two genomes had only 53% and 56% similarity to the 3 genomes clustering in the unnamed branch. Interestingly, one sample (PB10) harbored one virus genome belonging to each branch, the bavoviruses and the unnamed branch.

### 3.7. Smacoriridae

**Figure 7:**
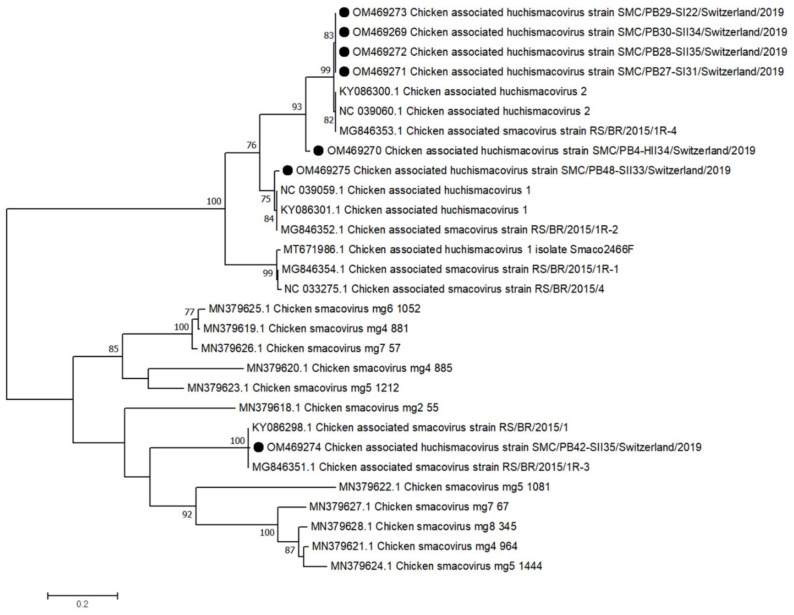
Phylogenetic tree based on the amino acid sequence identity of the Rep protein of selected chicken smaco/huchismacoviruses. *Huchismacovirus* contigs obtained in this study are marked with black dots. Phylogenetic analysis was performed using the maximum likelihood algorithm based on the Tamura–Nei model with the 1000 replication bootstrap method. Only values ≥ 70% were displayed. Evolutionary analyses were conducted using MEGA X.

The *Smacoviridae* belongs to the *Cressdnaviricota* and is often collectively called CRESS-DNA viruses. Smacoviruses have previously been discovered in metagenomics studies and are suspected to infect animals (ICTV, EC51, Berlin). Viral contigs belonging to *Smacoviridae*, genus *Huchismacovirus*, were constructed in 6 RSS flocks, 2 from the first (days 22 and 31) and 4 from the second (days 33–35) collection time point. The single *Smacovirus* contig constructed from a healthy flock was collected at day 34 and shows 90% Rep protein aa identity to 4 contigs from RSS flocks and to a reference strain of chicken-associated huchismacovirus 2 (NC039060) and 82% Rep aa identity to chicken-associated huchismacovirus 1 (NC099059). The 4 contigs from RSS flocks (OM469269 and OM469271-OM469273) share 99% Rep aa identity to chicken-associated huchismacovirus 2 (NC039060) and 76% to chicken-associated huchismacovirus 1 (NC099059). One huchismacovirus contig (OM469275) shows 97% aa identity to chicken-associated huchismacovirus 1 (NC099059). Finally, contig OM469274 showed only 28% and 31% Rep protein aa identity to the reference huchismacovirus 1 and 2 strains, respectively. The same contig shares 99% Rep aa identity with 2 unclassified *Smacovirus* strains from Brazil (KY086298 and MG846351) (Figure 7).

## 4. Discussion

High-throughput sequencing opened a new chapter in the characterization of the virome of various species and in the understanding of the ecology and evolution of specific viruses. The avian virome is not well characterized, especially that of healthy broiler chickens, as most prior studies focused on diseased or wild animals [19]. Including healthy broiler flocks, as in the present study, is crucial to uncovering potential culprits of RSS. As the virome likely changes over time, two time points for sample collection were included in our study.

RSS is a considerable economic problem in broiler chickens worldwide. Studies in recent years to identify the causative agent were limited mainly to PCR analysis of selected viruses. Heretofore, many viruses were associated with RSS, i.e., astroviruses, avian nephritis viruses, reoviruses, rotaviruses, and parvoviruses [7]. A recent metagenomic study on affected and unaffected broilers did not find any association between detected viruses and RSS; however, the study was limited by the low number of animals [8]. Since it is difficult to collect fecal samples from individual animals, we decided to collect samples from the entire flock, as it is collected routinely for *Salmonella* tests [20]. We are aware that this method can be biased because viruses persisting from a previous flock may contaminate the production facility even before the settlement of the new flock. However, relatively few viral reads were identified in the samples collected at the beginning of production (days 2–4), indicating clean environmental conditions and a low virus burden in introduced animals.

The ratio of viral reads relative to total reads ranged from an average of 0.5% (0.0003–1.92%) in healthy flocks at the beginning of production (days 2–11) to 1.38% (0.26–8.31%) in sick flocks sampled immediately after the onset of symptoms. Although the numbers of virus reads were low in the healthy animals at the first sampling time point, CAstV and ANV had the highest relative read abundance in that group and also when compared with all other groups investigated. Interestingly, these two viruses have been associated with growth problems and enteritis in young chickens already in the first week of live [21]. Astroviruses have an incubation period of 4–5 days; hence, new animals introduced to the breeding facility may lower immunity due to the stress level [22]. Virtually complete genomes of CAstV and ANV were constructed by de novo analysis of reads exclusively from the first sampling time point and showed high identity to each other independent of whether they originated from healthy or diseased animals, further indicating that these two viruses may not be the causative agents of RSS.

Viral sequences of members of the *Parvoviridae* were detected across all samples, with high abundance starting on day 8 and staying high until the last day of sample collection in both groups. Detection at the early time points is not uncommon, since previous studies suggested the possibility of vertical viral transmission [23,24]. Moreover, parvoviruses are broadly detected in healthy broilers [25,26,27]. The virus genomes detected in this study showed high similarities to both aveparvoviruses and depandoparvoviruses previously isolated from healthy broiler chicken [28,29]. The persistence of the virus in flocks may be correlated with hygiene measures, as indicated earlier by Zhang et al., who found that flocks kept in open production facilities were more frequently positive for parvoviruses than flocks kept in closed facilities. The most frequent viruses from the genus *Chaphamaparvovirus* have been recently detected by metagenomic analysis of many different vertebrates, including bats, dogs, cats, rodents, pigs, and birds, e.g., chickens, turkey, parrots, ducks, and pheasants [8,30,31,32,33,34,35,36,37,38]. While most studies report the detection of chaphamaparvoviruses in animals with enteritis and diarrhea, these viruses have also been detected in healthy animals [39,40]. Furthermore, Lima et al., reported no significant differences in chaphamaparvoviruses reads between healthy birds and birds with malabsorption syndrome [8]. This is supported by findings from our study, as chaphamaparvoviruses sequences were detected in all flocks. In total, we assembled 97 complete or near-complete genomes, and many flocks hosted up to 3 different strains. The 97 genomes clustered to either chaphamaparvoviruses 1 and 2, although 28 of the genomes found here showed only approximately 86–87% nt similarity to chaphamaparovivirus 2 and should be subclustered into a new chaphamaparovivirus subspecies, e.g., 2b. Interestingly, we have identified one healthy flock that harbored five different parvoviruses, including 3 chaphamaparvoviruses, 1 aveparovovirus, and 1 depandoparvovirus.

Viral reads belonging to *Picornaviridae* were found in 43 samples. A previous study showed that picornaviruses are significantly associated with histological lesions [7]. However, a metagenomic study reported that picornaviruses were the most abundant viruses in both healthy and sick chickens [41]. Picornaviruses are known for their high stability in the environment, although in our study, they were detected for the first time on day 7 in an RSS flock [42]. By comparing with other *Sicinivirus* strains, 21 genomes detected in our study share similarities to strains A1, A3, A5, and A8. Nevertheless, there was no clear difference between the sequences detected in healthy and RSS flocks. Twenty contigs assigned to the genus *Anativirus* were clustered together with the anativirus B1 strain Pf-CHK1/PhV detected in diarrheic chicken simultaneously co-infected with 6 avian picornaviruses classified to 6 different genera [43]. *Megrivirus* genomes present in our study were clustered in species C; 4 of the genomes showed high similarity to C1, and 8 genomes were assigned to their own branch, suggesting a new species. Megriviruses were identified in the proventriculars of chickens diagnosed with transmissible viral proventriculitis and were associated with hepatitis in turkeys [44,45]. In our study, megriviruses were mainly detected in RSS flocks at the end of the production and in one healthy sample at day 34. The last 4 picornavirus genomes belong to the genus *Gallivirus*, with one genome closely related to species A2, and 3 genomes sharing only 69% with A2, suggesting a new species. Galliviruses were previously detected in two consecutive flocks in the same building, indicating high stability, even with good hygiene [7]. Overall, picornaviruses are widely identified in chicken samples, with frequent co-infections with several different picornaviruses, which may lead to fast evolution, high diversity, and recombination events [46,47].

Reoviruses, such as ARVs and RVs, are proven pathogens associated with gastrointestinal diseases, malabsorption, and runting-stunting syndrome [48,49]. In this study, ARV and RVA were detected mainly in RSS flocks, indicating that reoviruses are a potential cause of RSS in at least some flocks. Previous studies have shown that RVs are usually not excreted in broilers younger than 14 days due to maternal immunity [49,50]. However, under poorer hygiene conditions and density stress, younger broilers may be more susceptible to RVs [50,51]. Nevertheless, two almost fully sequenced RVAs in our study are from sick flocks with 13- and 33-day-old broilers. The two genomes showed the same genotype constellation and approximately 90–97% nt similarities to the first fully sequenced RVA isolated from RSS-affected chicken fecal samples collected in 2002 in Germany [52]. As RVs are a major concern in broiler production, the only means of prevention is good hygiene management since no commercial vaccine is available. While in a previous metagenomic study on RSS-affected broilers, Lima et al., reported detection of mainly RVD and RVF and only one contig of RVA, we detected mainly RVAs, few sequences of RVF, and no RVD [8]. While ARVs were detected in RSS flocks within days 14–20 in our work, these viruses were not found in the previous study. It is important to point out that ARVs similar to RVs were detected from day 14 onwards, supporting the hypothesis of maternal immunity. Parvoviruses were detected as early as day 2 of production, but the abundance increased markedly after day 14.

Caliciviruses were previously identified in chicken flocks associated with RSS, as well as in asymptomatic flocks. The Bavaria/04V0021 strain was the first representative of the *Bavovirus* genus and originated from animals without clinical symptoms [53]. The two calicivirus genomes identified in this study were clustered in the same genus and originated from both RSS flocks and healthy flocks. All other calicivirus genomes detected here clustered in a new genus with strains recently found in the Netherlands (unpublished data; for GenBank acc. numbers see Figure 6), making the conclusion of a potential impact of caliciviruses for RSS difficult.

Smacoviruses belonging to the circular replication-initiation protein encoding single-stranded (CRESS) DNA viruses were broadly detected in fecal samples of various vertebrates [54,55]. In our study, we detected *Huchismacovirus* genomes that clustered together with other known chicken huchismacoviruses in almost all samples, with a higher abundance in older animals.

RSS is a multifactorial disease that, besides viral co-infection, also involves bacteria, environmental conditions, and the health status of the animals. At the end of the production, a high abundance of different viruses was present in both healthy and RRS animals. However, the presence of viruses in the ground feces does not necessarily correlate with the health status of all chickens within the flocks. Viruses in healthy flocks may cause asymptomatic or mild infections that remain undetected. Therefore, next to *Reoviridae*, we cannot exclude the potential role of caliciviruses and picornaviruses in RSS, as these viruses were found in healthy flocks at the end of production and cause subclinical infections. Further studies to determine their role in RSS should be conducted. The hatching facilities and chicken eggs should be investigated to determine the role of virus introduction into flocks.

## 5. Conclusions

The relative abundance of avian viruses was higher in RSS-affected broiler flocks at both time points. However, it is not possible to indicate a virus clearly associated with the disease. Although RVA and ARVs were detected mainly in RSS-affected broiler flocks, those findings were not consistent for all flocks. Some of the viruses detected in this study may have a commensal role in the avian virome or become pathogenic under certain conditions, i.e., poor hygiene management, low maternal immunity, or high animal density. Given that the virome of healthy and diseased broiler flocks both changed over time of production and that multiple co-infections occurred, it is difficult to define the role of each virus in health and disease.

## Figures and Tables

**Table 1:** Normalized number of viral reads detected in samples at two different sampling time points. The color gradient represents the numbers of viral reads from the 15 percentiles of the lowest number (white) to the 85 percentiles of the highest number (dark gray).

Virus Family	Total Healthy	1st Collection	2nd Collection	Total RSS	1st Collection	2nd Collection
*Parvoviridae*	5.1 × 10^−03^	2.7 × 10^−04^	4.8 × 10^−03^	1.6 × 10^−02^	1.3 × 10^−02^	3.8 × 10^−03^
*Astroviridae*	4.6 × 10^−03^	4.6 × 10^−03^	2.9 × 10^−06^	3.3 × 10^−04^	3 × 10^−04^	3.3 × 10^−05^
*Picornaviridae*	1.6 × 10^−03^	7.8 × 10^−05^	1.5 × 10^−03^	2.6 × 10^−03^	6.5 × 10^−04^	1.9 × 10^−03^
*Caliciviridae*	2.3 × 10^−05^	4.8 × 10^−06^	1.8 × 10^−05^	1.2 × 10^−04^	1.8 × 10^−05^	9.8 × 10^−05^
*Reoviridae*	4.8 × 10^−06^	3.6 × 10^−07^	4.5 × 10^−06^	2.7 × 10^−04^	1.4 × 10^−04^	1.3 × 10^−04^
*Adenoviridae*	2.7 × 10^−05^	1.2 × 10^−07^	2.3 × 10^−05^	1.7 × 10^−05^	6.2 × 10^−07^	1.6 × 10^−05^
*Coronaviridae*	-	-	-	1.4 × 10^−05^	-	1.4 × 10^−05^
*Smacoviridae*	2.5 × 10^−05^	1.4 × 10^−05^	1.1 × 10^−05^	1.6 × 10^−03^	5.1 × 10^−05^	1.5 × 10^−03^
*unknown*	1.5 × 10^−05^	1.3 × 10^−05^	1.9 × 10^−06^	8.7 × 10^−06^	6.7 × 10^−06^	2. × 10^−06^

**Table 2:** Heat map of normalized number of viral family reads detected in samples on different days of production. The following gradient is used: light gray: 15 percentiles of the lowest number; dark gray: 85 percentiles of the highest number of reads; white: viral reads not detected. HI, first sample collection at healthy flocks; HII, second sample collection at healthy flocks; SI, first sample collection at RSS-affected flocks; SII, second sample collection at RSS-affected flocks.

	Day	2	3	4	5	6	7	8	11	12	14	16	19	20	21	22	23	26	28	30	31	32	33		34		35		36
Virus		HI	HI	HI	HI	HI	SI	SI	HI	SI	SI	SI	SI	SI	SI	SI	SI	SI	HII	HII	SII	HII	HII	SII	HII	SII	HII	SII	HII
*Parvoviridae*																													
*Astroviridae*																													
*Picornaviridae*																													
*Caliciviridae*																													
*Reoviridae*																													
*Adenoviridae*																													
*Coronaviridae*																													
*Smacoviridae*																													
*unknown*																													

**Table 3:** Results from genotyping RVA segments from this study using the annotation pipeline available at https://www.viprbrc.org/.

Sequence Identifier	Gene Name	Genotype	Closest Strain	Identity %
OM469184	VP1	R6	DEU/02V0002G3/2002/G19P30	90.5
OM469183	VP1	R6	DEU/02V0002G3/2002/G19P30	90.5
OM469185	VP2	C6	DEU/02V0002G3/2002/G19P30	94.4
OM469186	VP2	C6	DEU/02V0002G3/2002/G19P30	94.3
OM469187	VP3	M7	DEU/02V0002G3/2002/G19P30	95
OM469188	VP3	M7	DEU/02V0002G3/2002/G19P30	95.2
OM469189	VP4	P [30]	DEU/02V0002G3/2002/G19P30	94.2
OM469190	VP4	P [30]	DEU/02V0002G3/2002/G19P30	94.1
OM469191	VP6	I11	DEU/02V0002G3/2002/G19P30	95.7
OM469192	VP6	I11	DEU/02V0002G3/2002/G19P30	95.6
OM469193	VP7	G19	XXX/Ch-1/XXXX/G19P17	96.4
OM469194	VP7	G19	XXX/Ch-1/XXXX/G19P17	94.9
OM469196	NSP1	A16	DEU/02V0002G3/2002/G19P30	97.8
OM469195	NSP1	A16	DEU/02V0002G3/2002/G19P30	97.9
OM469198	NSP2	N6	DEU/02V0002G3/2002/G19P30	97.4
OM469197	NSP2	N6	DEU/02V0002G3/2002/G19P30	94.9
OM469200	NSP3	T8	DEU/02V0002G3/2002/G19P30	89.6
OM469199	NSP3	T8	DEU/02V0002G3/2002/G19P30	89.5
OM469201	NSP4	E10	XXX/Ch-1/XXXX/G19P17	91.9
OM469202	NSP5/NSP6	H8	DEU/02V0002G3/2002/G19P30	93.8
OM469203	NSP5/NSP6	H8	DEU/02V0002G3/2002/G19P30	93.6

## Data Availability

The nucleotide sequences of viruses from this study shown in the phylogenetic analysis have been registered at GenBank under the following accession numbers: OM469021-OM469308. All raw sequencing data generated during this study were uploaded to the Sequence Read Archive (SRA) under accession number PRJNA802076.

## References

[1] Hafez H.M., Attia Y.A. (2020). Challenges to the poultry industry: Current perspectives and strategic future after the COVID-19 outbreak. Front. Vet. Sci..

[2] Clemente J.C., Ursell L.K., Parfrey L.W., Knight R. (2012). The impact of the gut microbiota on human health: An integrative view. Cell.

[3] Mihalov-Kovács E., Fehér E., Martella V., Bányai K., Farkas S.L. (2014). The fecal virome of domesticated animals. Virusdisease.

[4] Paez-Espino D., Eloe-Fadrosh E.A., Pavlopoulos G.A., Thomas A.D., Huntemann M., Mikhailova N., Rubin E., Ivanova N.N., Kyrpides N.C. (2016). Uncovering earth’s virome. Nature.

[5] Kouwenhoven B., Davelaar F.G., Van Walsum J. (1978). Infectious proventriculitis causing runting in broilers. Avian Pathol..

[6] Rebel J.M.J., Balk F.R.M., Post J., Van Hemert S., Zekarias B., Stockhofe N. (2006). Malabsorption syndrome in broilers. World’s Poult. Sci. J..

[7] De Oliveira L.B., Stanton J.B., Zhang J., Brown C., Butt S.L., Dimitrov K., Afonso C.L., Volkening J.D., Lara L.J.C., de Oliveira C.S.F. (2021). Runting and stunting syndrome in broiler chickens: Histopathology and association with a novel picornavirus. Vet. Pathol..

[8] Lima D.A., Cibulski S.P., Tochetto C., Varela A.P.M., Finkler F., Teixeira T.F., Loiko M.R., Cerva C., Junqueira D.M., Mayer F.Q. (2019). The intestinal virome of malabsorption syndrome-affected and unaffected broilers through shotgun metagenomics. Virus Res..

[9] Bovo S., Schiavo G., Bolner M., Ballan M., Fontanesi L. (2022). Mining livestock genome datasets for an unconventional characterization of animal DNA viromes. Genomics.

[10] Kwok K.T.T., Nieuwenhuijse D.F., Phan M.V.T., Koopmans M.P.G. (2020). Virus metagenomics in farm animals: A systematic review. Viruses.

[11] Kubacki J., Fraefel C., Bachofen C. (2021). Implementation of next-generation sequencing for virus identification in veterinary diagnostic laboratories. J. Vet. Diagn. Investig..

[12] Kubacki J., Flacio E., Qi W., Guidi V., Tonolla M., Fraefel C. (2020). Viral metagenomic analysis of aedes albopictus mosquitos from southern switzerland. Viruses.

[13] Hardmeier I., Aeberhard N., Qi W., Schoenbaechler K., Kraettli H., Hatt J.M., Fraefel C., Kubacki J. (2021). Metagenomic analysis of fecal and tissue samples from 18 endemic bat species in switzerland revealed a diverse virus composition including potentially zoonotic viruses. PLoS ONE.

[14] Bolger A.M., Lohse M., Usadel B. (2014). Trimmomatic: A flexible trimmer for illumina sequence data. Bioinformatics.

[15] Nurk S., Meleshko D., Korobeynikov A., Pevzner P.A. (2017). Metaspades: A new versatile metagenomic assembler. Genome Res..

[16] Altschul S.F., Gish W., Miller W., Myers E.W., Lipman D.J. (1990). Basic local alignment search tool. J. Mol. Biol..

[17] Muhire B.M., Varsani A., Martin D.P. (2014). Sdt: A virus classification tool based on pairwise sequence alignment and identity calculation. PLoS ONE.

[18] Kumar S., Stecher G., Li M., Knyaz C., Tamura K. (2018). Mega x: Molecular evolutionary genetics analysis across computing platforms. Mol. Biol. Evol..

[19] François S., Pybus O.G. (2020). Towards an understanding of the avian virome. J. Gen. Virol..

[20] Kingston D.J. (1981). A comparison of culturing drag swabs and litter for identification of infections with *Salmonella* spp. In commercial chicken flocks. Avian Dis..

[21] Zhang F., Li Y., Jiang W., Yu X., Zhuang Q., Wang S., Yuan L., Wang K., Sun S., Liu H. (2022). Surveillance and genetic diversity analysis of avian astrovirus in china. PLoS ONE.

[22] Lee R.M., Lessler J., Lee R.A., Rudolph K.E., Reich N.G., Perl T.M., Cummings D.A. (2013). Incubation periods of viral gastroenteritis: A systematic review. BMC Infect. Dis..

[23] Kisary J. (1985). Experimental infection of chicken embryos and day-old chickens with parvovirus of chicken origin. Avian Pathol..

[24] Kisary J., Miller-Faures A., Rommelaere J. (1987). Presence of fowl parvovirus in fibroblast cell cultures prepared from uninoculated white leghorn chicken embryos. Avian Pathol..

[25] Zsak L., Strother K.O., Day J.M. (2009). Development of a polymerase chain reaction procedure for detection of chicken and turkey parvoviruses. Avian Dis..

[26] Palade E.A., Kisary J., Benyeda Z., Mándoki M., Balka G., Jakab C., Végh B., Demeter Z., Rusvai M. (2011). Naturally occurring parvoviral infection in hungarian broiler flocks. Avian Pathol..

[27] Palade E.A., Demeter Z., Hornyák A., Nemes C., Kisary J., Rusvai M. (2011). High prevalence of turkey parvovirus in turkey flocks from hungary experiencing enteric disease syndromes. Avian Dis..

[28] Wang J., Zhu L., Zhu J., Sun H., Zhu G. (2011). Molecular characterization and phylogenetic analysis of an avian adeno-associated virus originating from a chicken in China. Arch. Virol..

[29] Estevez C., Villegas P. (2004). Sequence analysis, viral rescue from infectious clones and generation of recombinant virions of the avian adeno-associated virus. Virus Res..

[30] Baker K.S., Leggett R.M., Bexfield N.H., Alston M., Daly G., Todd S., Tachedjian M., Holmes C.E., Crameri S., Wang L.F. (2013). Metagenomic study of the viruses of african straw-coloured fruit bats: Detection of a chiropteran poxvirus and isolation of a novel adenovirus. Virology.

[31] Palombieri A., Di Profio F., Lanave G., Capozza P., Marsilio F., Martella V., Di Martino B. (2020). Molecular detection and characterization of carnivore chaphamaparvovirus 1 in dogs. Vet. Microbiol..

[32] Li Y., Gordon E., Idle A., Altan E., Seguin M.A., Estrada M., Deng X., Delwart E. (2020). Virome of a feline outbreak of diarrhea and vomiting includes bocaviruses and a novel chapparvovirus. Viruses.

[33] Yang S., Liu Z., Wang Y., Li W., Fu X., Lin Y., Shen Q., Wang X., Wang H., Zhang W. (2016). A novel rodent chapparvovirus in feces of wild rats. Virol. J..

[34] Palinski R.M., Mitra N., Hause B.M. (2016). Discovery of a novel parvovirinae virus, porcine parvovirus 7, by metagenomic sequencing of porcine rectal swabs. Virus Genes.

[35] Reuter G., Boros Á., Delwart E., Pankovics P. (2014). Novel circular single-stranded DNA virus from turkey faeces. Arch. Virol..

[36] Sarker S. (2021). Molecular and phylogenetic characterisation of a highly divergent novel parvovirus (psittaciform chaphamaparvovirus 2) in Australian neophema parrots. Pathogens.

[37] Vibin J., Chamings A., Klaassen M., Bhatta T.R., Alexandersen S. (2020). Metagenomic characterisation of avian parvoviruses and picornaviruses from Australian wild ducks. Sci. Rep..

[38] Matos M., Bilic I., Viloux N., Palmieri N., Albaric O., Chatenet X., Tvarogová J., Dinhopl N., Heidl S., Liebhart D. (2022). A novel chaphamaparvovirus is the etiological agent of hepatitis outbreaks in pheasants (*Phasianus colchicus*) characterized by high mortality. Transbound. Emerg. Dis..

[39] Fahsbender E., Altan E., Seguin M.A., Young P., Estrada M., Leutenegger C., Delwart E. (2019). Chapparvovirus DNA found in 4% of dogs with diarrhea. Viruses.

[40] Abayli H., Can-Sahna K. (2022). First detection of feline bocaparvovirus 2 and feline chaphamaparvovirus in healthy cats in Turkey. Vet. Res. Commun..

[41] Devaney R., Trudgett J., Trudgett A., Meharg C., Smyth V. (2016). A metagenomic comparison of endemic viruses from broiler chickens with runting-stunting syndrome and from normal birds. Avian Pathol..

[42] Rzezutka A., Cook N. (2004). Survival of human enteric viruses in the environment and food. FEMS Microbiol. Rev..

[43] Boros Á., Pankovics P., Adonyi Á., Fenyvesi H., Day J.M., Phan T.G., Delwart E., Reuter G. (2016). A diarrheic chicken simultaneously co-infected with multiple picornaviruses: Complete genome analysis of avian picornaviruses representing up to six genera. Virology.

[44] Kim H.R., Yoon S.J., Lee H.S., Kwon Y.K. (2015). Identification of a picornavirus from chickens with transmissible viral proventriculitis using metagenomic analysis. Arch. Virol..

[45] Boros Á., Pankovics P., Reuter G. (2014). Avian picornaviruses: Molecular evolution, genome diversity and unusual genome features of a rapidly expanding group of viruses in birds. Infect. Genet. Evol. J. Mol. Epidemiol. Evol. Genet. Infect. Dis..

[46] Boros Á., Pankovics P., Knowles N.J., Nemes C., Delwart E., Reuter G. (2014). Comparative complete genome analysis of chicken and turkey megriviruses (Family Picornaviridae): Long 3′ untranslated regions with a potential second open reading frame and evidence for possible recombination. J. Virol..

[47] Lau S.K.P., Woo P.C.Y., Yip C.C.Y., Li K.S.M., Fan R.Y.Y., Bai R., Huang Y., Chan K.H., Yuen K.Y. (2014). Chickens host diverse picornaviruses originated from potential interspecies transmission with recombination. J. Gen. Virol..

[48] Tang Y., Lu H., Sebastian A., Yeh Y.T., Praul C.A., Albert I.U., Zheng S.Y. (2015). Genomic characterization of a turkey reovirus field strain by next-generation sequencing. Infect. Genet. Evol..

[49] McNulty M.S., Allan G.M., McCracken R.M. (1983). Experimental infection of chickens with rotaviruses: Clinical and virological findings. Avian Pathol..

[50] Da Silva R.R., Bezerra D.A.M. (2013). Molecular epidemiology of avian rotavirus in fecal samples of broiler chickens in Amazon region, Brazil, from August 2008 to May 2011. Rev. Pan-Amaz. Saúde.

[51] Pantin-Jackwood M.J., Day J.M., Jackwood M.W., Spackman E. (2008). Enteric viruses detected by molecular methods in commercial chicken and turkey flocks in the United States between 2005 and 2006. Avian Dis..

[52] Trojnar E., Otto P., Johne R. (2009). The first complete genome sequence of a chicken group a rotavirus indicates independent evolution of mammalian and avian strains. Virology.

[53] Wolf S., Reetz J., Otto P. (2011). Genetic characterization of a novel calicivirus from a chicken. Arch. Virol..

[54] Varsani A., Krupovic M. (2018). Smacoviridae: A new family of animal-associated single-stranded DNA viruses. Arch. Virol..

[55] Simmonds P., Adams M.J., Benkő M., Breitbart M., Brister J.R., Carstens E.B., Davison A.J., Delwart E., Gorbalenya A.E., Harrach B. (2017). Consensus statement: Virus taxonomy in the age of metagenomics. Nat. Rev. Microbiol..

